# Signal Detection of Adverse Events Associated with Four Dihydropyridine Calcium Channel Blockers Based on the FAERS Database

**DOI:** 10.3390/ph19040544

**Published:** 2026-03-28

**Authors:** Zicong Guo, Yi Guo, Xiaoxiao Quan, Rui Xiao, Jia Li, Wei Liu

**Affiliations:** School of Pharmaceutical Sciences, Zhengzhou University, No. 100 Science Avenue, High-Tech Zone, Zhengzhou 450001, China; guozicongyxy@gs.zzu.edu.cn (Z.G.); lyqxx88@163.com (X.Q.); xiaoyyy@gs.zzu.edu.cn (R.X.); li15837016326@gs.zzu.edu.cn (J.L.)

**Keywords:** DHP-CCB, FAERS, real-world study, adverse events, pharmacovigilance

## Abstract

**Objectives**: As widely used first-line antihypertensive drugs, dihydropyridine calcium channel blockers (DHP-CCBs) have relatively few studies comparing their adverse reactions based on real-world data. This study aims to identify and compare the potential adverse drug reaction (ADR) signals of four DHP-CCBs (amlodipine, felodipine, nicardipine, and nifedipine) through the US Food and Drug Administration Adverse Event Reporting System (FAERS), providing a reference for further drug safety assessment and clinical medication risk awareness. **Methods**: Adverse event reports from medical professionals (Q3 2014–Q4 2024) were analyzed using signal detection methods, including reporting odds ratio (ROR), proportional reporting ratio (PRR), information component (IC), and the Medicines and Healthcare Products Regulatory Agency (MHRA) methods. Risk signals for the four DHP-CCBs were compared with both the full database and the DHP-CCBs background. For high-risk signals in amlodipine, multivariate logistic regression was used for validation. The analysis reveals distinct ADR profiles for the four DHP-CCBs. **Results**: Amlodipine is strongly linked to suicide-related risks, confirmed by logistic regression. Nicardipine and nifedipine show significant risks for pregnancy-related events, such as premature delivery and exposure during pregnancy. Nicardipine is also associated with hyponatremia, hyperkalemia, and lactic acidosis. These adverse events are not yet included in the FDA labeling for any of the DHP-CCBs. Although palpitations and angioedema are listed for felodipine, their signal strength is much higher compared to the other DHP-CCBs. **Conclusions**: The ADR risk profiles of the four DHP-CCBs differ significantly. This study identified several high-risk adverse events not included in current labels. Clinical use should consider each drug’s risk profile and patient-specific factors, with particular attention to serious risk signals. For pregnant and postpartum women, the benefits and risks of using nicardipine and nifedipine should be carefully evaluated.

## 1. Introduction

Hypertension is one of the most common chronic non-communicable diseases worldwide and is one of the most significant risk factors for major diseases such as ischemic heart disease, stroke, chronic kidney disease, and dementia [1]. Over the past three decades, the global prevalence of hypertension has remained consistently high, especially in low- and middle-income countries where it has continued to rise [2]. According to the World Health Organization (WHO) report in 2024, approximately 1.4 billion adults aged 30 to 79 years worldwide have hypertension, accounting for about 33% of the total population in this age group [3].

Dihydropyridine Calcium Channel Blockers (DHP-CCBs) are recommended as first-line or preferred agents for the treatment of hypertension, angina pectoris, and related cardiovascular conditions in multiple authoritative guidelines, owing to their favorable characteristics such as gradual onset of action, reliable antihypertensive efficacy, minimal effects on metabolism and renal function, and good overall tolerability [4,5]. They have maintained an important position in clinical practice for decades. The WHO has also included them in the Essential Medicines List. However, CCB drugs also have some well-defined Adverse Drug Reactions (ADRs), such as peripheral edema, hypotension, and tachycardia. In recent years, some studies have conducted safety signal mining for certain CCB drugs based on the US Food and Drug Administration Adverse Event Reporting System (FAERS) database. Jiang et al. [6] analyzed the adverse reaction signals related to amlodipine in the FAERS database using the proportional reporting ratio method. Chiappini et al. [7] explored the characteristics and occurrence frequency of adverse events related to the combined use of CCBs and cocaine based on the FAERS database. Despite this, systematic safety evaluation studies of DHP-CCBs using real-world big data are still limited, especially in terms of comparative analysis of the adverse reaction profiles among different drugs and specialized signal mining based on reports submitted by healthcare professionals in the FAERS database. Therefore, this study, relying on the FAERS database, extracted reports submitted by physicians, pharmacists, and health professionals, and selected four commonly used DHP-CCBs, namely amlodipine, felodipine, nicardipine, and nifedipine, to conduct in-depth mining and systematic comparison of their ADR signals, with the aim of providing more targeted references for rational clinical drug use.

## 2. Results

### 2.1. Basic Information of Adverse Event Reports for Four DHP-CCB Drugs

A total of 34,664 adverse events were reported for the four DHP-CCB drugs between the third quarter of 2014 and the fourth quarter of 2024. Amlodipine had the highest number of adverse event reports at 29,555 cases (85.26%). Female patients accounted for a significantly higher proportion than males (females: 19,131, 55.19%; males: 15,559, 44.89%). Adverse events associated with amlodipine, felodipine, and nicardipine primarily occur in middle-aged and elderly patients aged 44 years and older; nifedipine, however, is more commonly associated with adverse events in individuals aged 18 to 64 years. America reported the highest number of cases (10,870, 31.36%). Regarding reporting sources, physicians constituted the primary reporting entity, submitting the vast majority of adverse event reports. Patient outcomes reported were predominantly “other serious or important medical events” (39.21%) and “hospitalization-initial or prolonged” (33.46%), collectively exceeding 70%; ‘death’ and “life-threatening” events accounted for 14.59% and 6.95%, respectively. Detailed data are presented in Table 1.

### 2.2. Signal Analysis

**Figure 1:**
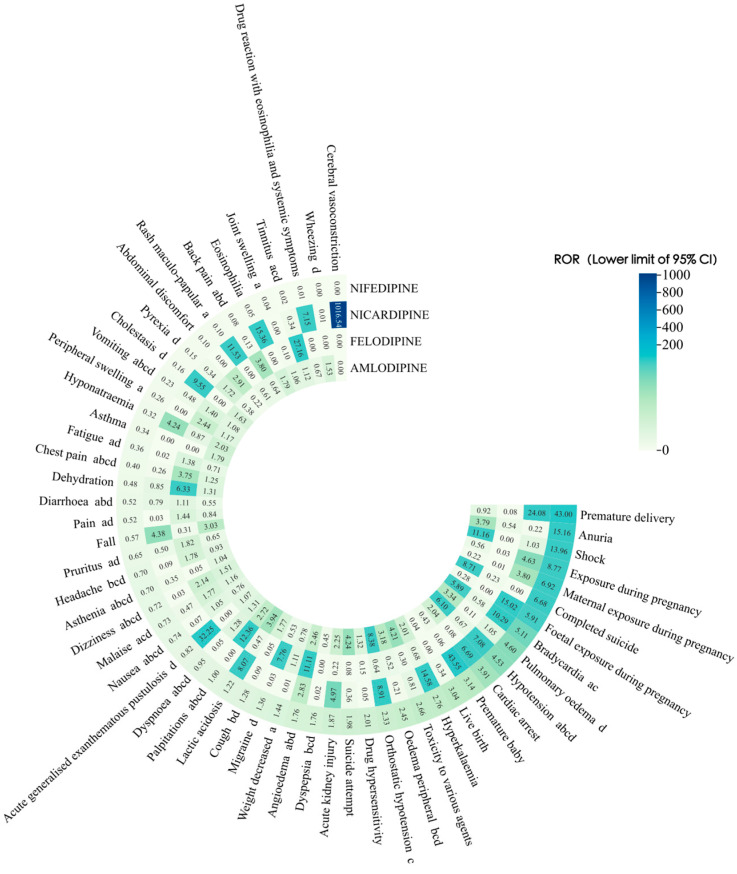
Comparison of ROR-Based Signal Detection Results (Lower 95% CI > 1) for Four DHP-CCB Drugs under the Background of the Entire Dataset. Note: a, b, c, and d indicate that the PT is included in the prescribing information for amlodipine, felodipine, nicardipine, and nifedipine, respectively.

**Figure 2:**
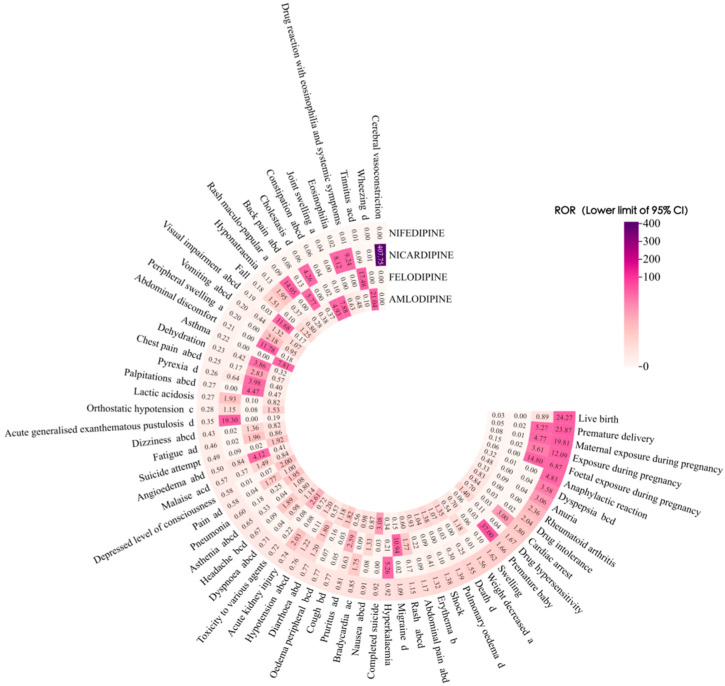
Comparison of ROR-Based Signal Detection Results (Lower 95% CI > 1) for Four DHP-CCB Drugs in the Context of DHP-CCBs. Note: a, b, c, and d indicate that the PT is included in the prescribing information for amlodipine, felodipine, nicardipine, and nifedipine, respectively.

This study conducted a proportional imbalance analysis of adverse events for four DHP-CCBs based on the complete dataset. The top 20 most frequent PTs for each drug were screened, and drug-unrelated adverse events such as drug interactions and off-label use were excluded, ultimately yielding 56 PTs. The risk signals identified by the four detection methods are shown in Table 2 and Table 3. ROR, PRR, and IC showed highly overlapping signal detection results, while the MHRA method identified fewer signals than the other three approaches. To visually represent signal detection outcomes and clarify signal intensity distribution, we visualized ROR-based results using a heatmap in which color depth is proportional to signal magnitude—darker shades indicate stronger signals. The details are shown in Figure 1. To further investigate the variability among target drugs, we separately compared the signal results for each target drug in the context of DHP-CCBs. Specific results are shown in Figure 2. The list of DHP-CCBs drugs is provided in Table S1.

The signal detection results across two different backgrounds revealed that, under the full background, both amlodipine and nifedipine exhibited significant signals for the PTs of completed suicide and suicidal attempt, with amlodipine showing a higher signal strength than nifedipine. As the detection background narrowed, only amlodipine continued to show effective signals for these two PTs, suggesting a significant statistical association between amlodipine and these adverse events. Additionally, under both detection backgrounds, amlodipine was the only drug that displayed effective signals for asthma, wheezing, and joint swelling, and these signals intensified significantly as the detection background narrowed. Notably, joint swelling is already included in amlodipine’s prescribing information.

To assess the association between four DHP-CCB drugs and completed suicide and suicide attempt, while controlling for potential confounding factors, we conducted a multivariate logistic regression analysis. The independent variables included drug type (amlodipine, felodipine, nicardipine, and nifedipine), gender (male, female), age group (<18, 18–44, 45–64, 65–75, >75), and the reporter’s occupation (physician, pharmacist, medical professional). Amlodipine, male, 18–44 years old, and physician were set as the reference group, and the adjusted odds ratios (aOR), 95% confidence intervals, and *p*-values of each independent variable were calculated. This method can more accurately assess the association between amlodipine and completed suicide when controlling for major confounding factors. The results are shown in Table S2. The results indicate that after adjusting for confounding factors such as age, gender, and reporter’s occupation, the rates of completed suicide and suicide attempt reports for amlodipine were significantly higher than those for the other three dihydropyridine drugs, which is consistent with the signal detection results of the imbalance ratio. In addition, to further address potential confounding, particularly from concomitant antidepressants and antipsychotics, a sensitivity analysis was conducted by excluding reports involving these medications. As shown in Supplementary Figure S1, the signals for completed suicide and suicide attempt associated with amlodipine remained significant after exclusion, supporting the robustness of the observed association.

Under both detection backgrounds, felodipine consistently showed high-intensity signals for PTs such as tinnitus, palpitations, angioedema, migraine, and dehydration. Under the full background, both amlodipine and felodipine showed effective signals for tinnitus, palpitations, and dehydration, but the signal strength was notably higher in felodipine. As the detection background narrowed, only felodipine continued to show effective signals for these PTs, with signal strength remaining relatively high. Migraine exhibited effective signals in both felodipine and nifedipine under the full background, but the signal strength in nifedipine was significantly lower than in felodipine. Under the background of all DHP-CCBs, only felodipine continued to show effective signals. For angioedema, effective signals were detected for all four target drugs under the full background, but the signal strength was significantly higher for felodipine compared to the other three DHP-CCBs. Furthermore, as the detection background narrowed, only felodipine exhibited an effective signal. Tinnitus, migraine, and dehydration are currently not included in felodipine’s prescribing information.

Four signal detection methods consistently indicated that nicardipine exhibited high-intensity signals for PTs such as cerebral vasoconstriction, premature baby, acute generalized exanthematous pustulosis (AGEP), premature delivery, eosinophilia, rash maculo-papular, and foetal exposure during pregnancy. These PTs are not currently included in nicardipine’s prescribing information. Compared with the other three target drugs, nicardipine displayed unique statistical associations with multiple PTs, including cerebral vasoconstriction, premature baby, AGEP, eosinophilia, drug reaction with eosinophilia and systemic symptoms (DRESS), rash maculo-papular, cholestasis, hyponatraemia, lactic acidosis, acute kidney injury (AKI), hyperkalemia, bradycardia, cardiac arrest, and foetal exposure during pregnancy. With the exception of bradycardia, all other PTs have not been included in nicardipine’s FDA prescribing information.

Among these, cerebral vasoconstriction, DRESS, and rash maculo-papular were the only PTs to form effective signals for nicardipine across both detection backgrounds. Eosinophilia, cholestasis, hyponatremia, and AGEP showed effective signals in both amlodipine and nicardipine under the full background, with nicardipine exhibiting significantly stronger signals compared to amlodipine. However, under the background of all DHP-CCBs, only nicardipine continued to show effective signals. Lactic acidosis, AKI, hyperkalemia, and bradycardia exhibited effective signals across all drugs, except for felodipine, with nicardipine showing stronger signals than amlodipine and nifedipine. However, only nicardipine continued to display effective signals under the background of all DHP-CCBs.

Under both detection backgrounds, nifedipine and nicardipine showed signals for premature baby and foetal exposure during pregnancy, with nicardipine exhibiting higher signal strength. Nifedipine and nicardipine both demonstrated signals for PTs such as premature delivery, exposure during pregnancy, and maternal exposure during pregnancy, which were not observed with the other two drugs, and these signals were stronger. However, the signal strength for nifedipine was significantly higher than that for nicardipine. Additionally, nifedipine also showed a statistical association with the PT of anuria. These PTs are not included in nifedipine’s prescribing information.

To minimize the potential influence of indication bias, a sensitivity analysis was performed for pregnancy-related adverse event signals of nicardipine and nifedipine. Reports in which the drug role was identified as primary suspect (PS) were retained, while reports explicitly indicating pregnancy-related conditions—such as gestational hypertension, preeclampsia, eclampsia, or indications related to tocolysis or pregnancy maintenance—were excluded. Signal detection was then repeated using the filtered dataset. As shown in Supplementary Figure S2, the pregnancy-related signals for nicardipine and nifedipine remained notable after excluding these reports and potential confounding from other drugs, further supporting the robustness of the study findings.

To further assess the potential influence of reporting behavior on the detected signals, a sensitivity analysis stratified by reporter type was conducted. Specifically, reports submitted by physicians were selected for re-analysis to improve data reliability and reduce potential reporting bias. The results of this sensitivity analysis are presented in Supplementary Figure S3. Notably, the overall findings were largely consistent with those of the primary analysis, indicating that the observed signals were not substantially affected by reporter type. These results further support the robustness of our findings.

These signal detection results highlight significant differences in the safety profiles of the four DHP-CCB drugs, indicating that each drug may carry varying risks for specific adverse events.

## 3. Discussion

This study systematically evaluated adverse events associated with amlodipine, felodipine, nicardipine, and nifedipine using the FAERS database. The study period was defined from 2014 Q3 to December 2024, as the FAERS database has consistently included the prod_ai (active ingredient) variable since 2014 Q3, enabling reliable active ingredient–level analyses. The end date corresponds to the most recent FAERS data available at the time of the study. Analysis incorporated reports submitted by physicians, healthcare professionals, and pharmacists to ensure quality. Signal detection was performed both across all drugs and specifically within the dihydropyridine class to exclude disease interference, enabling precise identification of drug-specific safety signals. We conducted a multivariate logistic regression analysis on the high-risk signals related to amlodipine—completed suicide and suicide attempt. The main considerations were as follows: The number of reports of amlodipine in the database was relatively sufficient, which could support further statistical analysis; at the same time, completed suicide and suicide attempt, as serious adverse mental events, might be interfered by confounding factors such as age and gender, and needed to be corrected through a multivariate model.

In addition, several sensitivity analyses were conducted to evaluate the robustness of the findings. These included excluding reports with pregnancy-related indications to minimize potential confounding effects on the signals observed for nicardipine and nifedipine, excluding reports involving concomitant use of antidepressants or antipsychotics to reduce their potential impact on suicide-related signals associated with amlodipine, and performing a stratified analysis by reporter type. The results of these sensitivity analyses were generally consistent with those of the primary analysis, supporting the robustness of the findings and enhancing the overall reliability of this study.

### 3.1. Analysis of Basic Report Information

Amlodipine and nifedipine are widely used for hypertension and angina, with much higher clinical use—and thus more FAERS reports—than nicardipine and felodipine. Female patients accounted for a greater proportion of adverse event reports, likely due to a higher proportion of elderly women, greater sensitivity to certain side effects, and more active reporting. These medications are commonly prescribed for middle-aged and elderly hypertensive patients, particularly those with atherosclerosis and coronary heart disease, explaining the higher reporting frequency within this demographic. Nifedipine’s rapid onset and short duration also make it more common in younger or acute cases, increasing reports among patients aged 18–44.The United States had the most reports, likely due to its large drug market, robust reporting system, high participation from healthcare professionals, and the FAERS being managed by the FDA. Physicians are the main reporters because of their expertise, clinical responsibility, and patient interactions. The severe outcomes associated with DHP-CCBs—such as hospitalization, life-threatening events, or death—may relate to the drugs’ pharmacologic effects, patient health status, reporting bias, and possible drug-related complications.

### 3.2. Analysis of Signal Detection Results

The high-frequency signals detected for amlodipine in this study align with the product label, including hypotension (ROR: 6.31, 95% CI: 6.10–6.54), bradycardia (ROR: 6.26, 95% CI: 5.89–6.65), dizziness (ROR: 1.60, 95% CI: 1.51–1.69), dyspnea (ROR: 1.38, 95% CI: 1.31–1.45) in the full background, and joint swelling under dihydropyridine background (ROR: 12.28, 95% CI: 7.88–19.15), constipation (ROR: 7.19, 95% CI: 4.93–10.50), and others, all of which are adverse reactions documented in the amlodipine prescribing information. This also demonstrates the reliability of our detection results. Concurrently, we identified several PTs not mentioned in the prescribing information but exhibiting high reporting frequency and signal strength, warranting further attention.

Signal detection results consistently showed that suicide attempt (ROR: 4.54, 95% CI: 4.24–4.87) and completed suicide (ROR: 9.00, 95% CI: 8.71–9.29) had stronger signals for amlodipine. As an L-type calcium channel blocker, amlodipine may induce psychiatric effects such as depression, leading to increased reports of suicidal behavior [8]. A recent FAERS analysis identified amlodipine among the top 20 drugs most frequently linked to “suicide and self-harm” events, with a notably high ROR for completed suicides [9]. Similarly, the American Association of Poison Control Centers reported that cardiovascular drugs, especially calcium channel blockers, are common agents in fatal overdoses [10]. Considering its wide use and easy access, the association between amlodipine and suicide attempts is understandable.

This study detected strong signals for PT-wheezing (ROR: 56.22, 95% CI: 21.04–150.19) and asthma (ROR: 11.72, 95% CI: 7.81–17.58) in the context of dihydropyridine calcium channel blockers. Excessive use of amlodipine or severe adverse reactions may induce non-cardiogenic pulmonary edema, presenting as respiratory distress and hypoxemia accompanied by dyspnea, moist rales, and wheezing, which may be reported as “wheezing/asthma” [11]. Amlodipine may also cause angioedema, characterized by painful swelling of the face, oropharynx, and respiratory tract [12]. If involving the pharynx, trachea, or bronchi, it can cause airway narrowing, edema, and respiratory obstruction, producing wheezing/asthma-like symptoms. Currently, there is no conclusive evidence that amlodipine directly causes asthma/wheezing, and further research is needed to validate this hypothesis.

Multiple PTs with high signal strength for felodipine have been included in its FDA labeling, including palpitations in all settings (ROR: 14.20, 95% CI: 12.36–16.31), angioedema (ROR: 13.06, 95% CI: 11.11–15.35), chest pain (ROR: 4.40, 95% CI: 3.75–5.17), dizziness (ROR: 2.48, 95% CI: 2.14–2.88), headache (ROR: 2.05, 95% CI: 1.78–2.35), and back pain in the dihydropyridine context (ROR: 7.07, 95% CI: 5.77–8.65), confirming the reliability of the findings.

Notably, in both analytical contexts, the signal strength for felodipine-associated palpitations and angioedema was significantly higher than the other three drugs. Although these two PTs are mentioned in the prescribing information, clinicians should remain vigilant and be alert to the potential risks of palpitations and angioedema when using felodipine. Additionally, we identified several PTs with significant signals that were not mentioned in the prescribing information, suggesting the potential for new adverse reactions warranting further investigation. Migraine (ROR: 8.93, 95% CI: 7.76–10.28) also exhibits a strong signal, potentially related to drug properties: Felodipine, a dihydropyridine calcium channel blocker, can cause peripheral and cerebral vasodilation, triggering the trigeminal-vascular system and releasing neuropeptides such as CGRP, thereby inducing headache or migraine-like symptoms [13]. The signal intensity for felodipine-related dehydration (ROR: 7.44, 95% CI: 6.33–8.74) was also notably high. Existing research suggests this phenomenon may be associated with the drug’s potential “direct tubular action,” meaning felodipine may promote sodium excretion. This diuretic sodium-excreting effect may entail water co-excretion, potentially leading to reduced body fluid volume [14]. This could represent a potential mechanism for the elevated dehydration signal intensity associated with felodipine. While these signals suggest a possible association between felodipine and these PTs, further studies are required to confirm this association.

Among the top twenty most frequently reported PTs for nicardipine, bradycardia (ROR: 13.54, 95% CI: 10.29–17.81) and hypotension (ROR: 8.61, 95% CI: 7.08–10.47) have been explicitly listed as adverse reactions by the FDA. However, analysis revealed numerous PTs with high signal strength that remain unlisted in the prescribing information. Particularly within the categories “Injuries, poisonings, and procedural complications,” “Pregnancy, puerperium and perinatal conditions,” and “Skin and subcutaneous tissue disorders,” a substantial number of PTs exhibited elevated signal strength. This suggests a potential association between these adverse events and nicardipine use, warranting further attention and investigation.

Cerebral vasoconstriction (ROR: 1361.91, 95% CI: 1016.54–1824.64) showed the strongest signal among nicardipine-related adverse events. This may result from two factors: first, nicardipine’s peripheral vasodilation could indirectly trigger cerebral vasoconstriction [15]; second, its clinical use in aneurysmal subarachnoid hemorrhage (aSAH) management [16]. Cerebral vasospasm, a common complication of aSAH and a form of cerebral vasoconstriction, typically occurs 3–14 days after bleeding [17].

Nicardipine shows strong signals for premature baby (ROR: 62.09, 95% CI: 43.55–88.53) and premature delivery (ROR: 32.44, 95% CI: 24.08–43.70). Nicardipine, widely used to treat gestational hypertension and preeclampsia [18], lowers blood pressure by inhibiting L-type calcium channels and inducing vasodilation. However, rapid or excessive blood pressure reduction may impair uteroplacental perfusion, leading to placental dysfunction, fetal hypoxia, and a higher risk of preterm birth or placental abruption. In a retrospective study, 35.8% (297/830) of nicardipine-treated pregnant women underwent emergency cesarean section for fetal distress, with one case directly linked to maternal hypotension two hours post-administration [19]. Under the “Injuries, poisoning and procedural complications” system classification, nicardipine-related adverse events included foetal exposure during pregnancy (ROR: 21.80, 95% CI: 15.02–31.64), exposure during pregnancy (ROR: 6.30, 95% CI: 4.63–8.57), and maternal exposure during pregnancy (ROR: 4.99, 95% CI: 3.80–6.54) were reported with higher frequency and generally significant signal strength. This phenomenon may be closely related to nicardipine’s widespread use in treating gestational hypertension [20].

Nicardipine exhibits a significant risk signal for AGEP (ROR: 45.25, 95% CI: 32.25–63.48). AGEP is a drug-induced T-cell-mediated hypersensitivity reaction, with its mechanism involving drug activation of specific T cells, release of inflammatory cytokines, and subsequent neutrophil infiltration [21]. Other CCBs (e.g., nifedipine) have reported cases of inducing AGEP [22], providing class-based evidence supporting nicardipine’s risk signal. Additionally, nicardipine exhibits strong signals for eosinophilia (ROR: 20.62, 95% CI: 15.36–27.69) and maculopapular rash (ROR: 15.95, 95% CI: 11.53–22.06). The potential mechanism may involve T-cell-mediated delayed-type hypersensitivity reactions. Similar reactions have been observed with other calcium channel blockers, suggesting possible structure-related immunological cross-reactivity [23,24].

This study found that nicardipine was also associated with cardiac arrest (ROR: 9.10, 95% CI: 6.69–12.39), AKI (ROR: 6.15, 95% CI: 4.97–7.61), hyponatremia (ROR: 5.97, 95% CI: 4.24–8.42), hyperkalemia (ROR: 18.95, 95% CI: 14.58–24.63), and lactic acidosis (ROR: 11.56, 95% CI: 8.07–16.57). These adverse events may constitute an interconnected pathophysiological process: nicardipine overdose or potent hypotension may trigger severe hypotension and inadequate tissue perfusion, leading to lactic acidosis [25]. Existing studies indicate a clear association between hypotension and AKI risk, with reports of increased AKI risk associated with high-dose nicardipine use in critical care/stroke settings [26]. Once AKI develops, impaired tubular reabsorption and secretion functions can trigger hyponatremia and hyperkalemia. Concurrent hyperkalemia with systemic hypoperfusion may further induce severe arrhythmias, potentially leading to cardiac arrest. Case reports document progressive progression from hypotension to AKI, severe hyperkalemia, and cardiac arrest due to potentiated calcium channel blocker effects [27]. These signals suggest a potential association between nicardipine and this sequence of events, warranting heightened clinical vigilance during therapeutic use.

Similarly to nicardipine, nifedipine also showed multiple strong signals for PT under the system classification “Injuries, Poisonings, and Surgical Complications,” including exposure during pregnancy (ROR: 9.88, 95% CI: 8.77–11.15) and maternal exposure during pregnancy (ROR: 7.70, 95% CI: 6.92–8.57). Multiple clinical practice guidelines currently recommend oral nifedipine as first-line therapy for acute severe hypertension in pregnancy [28], which may contribute to its prominent signals. From a pharmacokinetic perspective, studies confirm nifedipine crosses the placenta, with umbilical cord blood to maternal plasma concentration ratios ranging from 0.59 to 0.93, demonstrating effective placental transfer [29]. Additionally, a highly significant signal was detected for preterm delivery (ROR: 48.49, 95% CI: 43.00–54.69). Nifedipine is commonly used to inhibit preterm contractions and delay delivery [30], potentially postponing preterm birth by at least 48 h. However, since the treatment population inherently comprises high-risk preterm birth cases, treatment failure (i.e., inability to halt preterm labor progression) may also be recorded as “premature delivery occurring after drug administration.” Furthermore, nifedipine efficacy may be influenced by genetics (e.g., CYP3A5 polymorphism) [31], gestational hormones [32], and other clinical factors (e.g., maternal age, contraction intensity). Treatment failure due to insufficient drug effect in some patients directly contributes to the amplification of this signal.

Anuria (ROR: 48.49, 95% CI: 43.00–54.69) exhibited a high signal value in nifedipine. Studies indicate that CCBs block L-type calcium channels in the detrusor muscle, interfering with bladder contraction and impairing bladder emptying and filling functions. This may lead to urge incontinence, and severe lower urinary tract symptoms have been shown to correlate significantly with nifedipine [33]. Clinicians should be vigilant about this risk during clinical use.

## 4. Materials and Methods

### 4.1. Research Design and Data Sources

This study conducted a real-world pharmacovigilance retrospective analysis based on the FAERS database. FAERS is a spontaneous reporting system used by the FDA to monitor the safety of marketed drugs and therapeutic biologics. We extracted data from the FAERS database from the third quarter of 2014 to the fourth quarter of 2024. Duplicate reports were removed according to the FDA-recommended data processing approach. Specifically, records from the DEMO table were used for de-duplication based on the variables primaryid, caseid, and fda_dt. If multiple reports shared the same caseid, only the report with the most recent fda_dt (FDA receipt date) was retained. If both caseid and fda_dt were identical, the report with the largest primaryid was retained. This procedure ensured that only the most recent version of each case report was included in the analysis, thereby avoiding duplicate counting caused by follow-up submissions. Records with missing key information were excluded. Drug records were then filtered according to the role_cod variable in the DRUG table. In this study, three categories were included: PS (Primary Suspect), indicating the drug considered by the reporter to be the primary suspected cause of the adverse event; SS (Secondary Suspect), indicating a drug suspected to have contributed to the adverse event but not considered the primary cause; and I (Interacting), indicating a drug that may interact with the suspected drug and potentially contribute to the occurrence of the adverse event. We further filtered reports based on reporter occupation, retaining reports submitted by pharmacist, physician, and healthcare professional. After applying these criteria, a total of 3,657,573 reports were included as the complete dataset. To reduce the interference of underlying diseases and compare the adverse reaction characteristics of the four target drugs, we extracted the reports of DHP-CCBs as the control subset. DHP-CCBs correspond to the “selective calcium channel blockers with mainly vascular effects (C08C)” category under the Anatomical Therapeutic Chemical (ATC) classification system, specifically the “Dihydropyridine derivatives (C08CA)” active ingredients.

### 4.2. Selection of Target Drugs

In this study, the pharmacological category of the target drugs was dihydropyridine calcium channel blockers. The following target drugs were selected: amlodipine, felodipine, nicardipine, and nifedipine. All reports containing the generic names and brand names were extracted.

### 4.3. Coding and Standardization of Adverse Events

This study used the Preferred term (PT) and system organ classes (SOC) of the 28.0 version of the Medical Dictionary for Regulatory Activities (MedDRA) [34] to standardize and classify the descriptions of adverse events using international medical terminology.

### 4.4. Statistical Methods

This study employed four disproportionality methods, including reporting odds ratio (ROR), proportional reporting ratio (PRR), information component (IC), and the Medicines and Healthcare Products Regulatory Agency (MHRA), for drug ADR signal detection. A 2 × 2 table was constructed to obtain ADR data for the target drugs and non-target drugs, and based on the data in the table, the signal values for each method were calculated using the formulas. The signal detection thresholds were defined as follows: for the ROR and PRR methods, a signal was considered present when the number of reports (N) ≥ 3 and the lower limit of the 95% confidence interval (95% CI) > 1; for the MHRA method, a signal was defined when N ≥ 3, PRR ≥ 2, and χ^2^ ≥ 4; and for the IC method, a signal was considered present when N ≥ 3 and the lower limit of the 95% confidence interval of IC (IC025) > 0. The specific calculation formulas and signal determination criteria are shown in Table S3. To improve the robustness and reliability of signal detection and reduce potential false-positive findings, an ADR was considered to generate an effective signal only when it simultaneously met the signal detection criteria across all four methods. The study used R software (Version 4.1.3) for data processing and analysis, and the signal detection results of the ROR method were visualized in a heatmap. This study first conducted a disproportionality analysis based on the entire dataset to explore the common risks of the four DHP-CCB drugs, and then performed subset analysis in the context of DHP-CCBs to compare the ADR characteristics among the four drugs.

## 5. Conclusions

This study, based on real-world adverse event signal detection, identified statistical signals of disproportionate reporting between four DHP-CCBs—amlodipine, felodipine, nicardipine, and nifedipine—and several high-risk PTs not yet covered in the current FDA labeling. For instance, statistical signals suggested potential associations between amlodipine and psychiatric adverse events such as completed suicide and suicide attempt; between felodipine and tinnitus and migraine; between nicardipine and various neurological disorders (e.g., cerebral vasoconstriction), dermatological and subcutaneous tissue disorders (e.g., AGEP, DRESS, and rash maculo-papular), as well as pregnancy-related adverse events (e.g., premature delivery, foetal exposure during pregnancy); and between nifedipine and anuria and pregnancy-related adverse events (e.g., exposure during pregnancy, maternal exposure during pregnancy). Furthermore, although some severe adverse events (e.g., palpitations and angioedema associated with felodipine, bradycardia associated with amlodipine, nicardipine, and nifedipine) are already included in the drug labels, this study detected high-frequency and high-intensity reporting signals, indicating disproportionate reporting and suggesting that continued clinical vigilance may be warranted.

This study systematically demonstrated distinct safety-related statistical patterns among the four DHP-CCBs and, in conjunction with the existing literature, preliminarily discussed the potential mechanisms that may underlie these observed statistical associations. These results represent hypotheses-generating signals rather than evidence of causal relationships, and highlight the need for further well-designed pharmacoepidemiological studies to verify these signals, evaluate potential causality, and clarify underlying biological pathways. While the current findings are insufficient to establish causal relationships or directly inform changes in clinical practice, they provide an initial signal for clinical monitoring and underscore the importance of considering potential risks when using these medications.

This study has certain limitations: (1) The data of this study is derived from the FAERS database. Although this database provides valuable information on drug safety, as a spontaneous reporting system, its data may have issues such as missing, duplicate and underreported cases, which may to some extent affect the reliability of the results; (2) Proportionality imbalance analysis can effectively detect potential safety signals, but due to the lack of exposure data, underreporting and selective bias, proportionality imbalance signals themselves cannot be interpreted as conclusive scientific evidence of a causal relationship between drugs and adverse events [35]. (3) In addition, the distribution of reports in the FDA Adverse Event Reporting System may be influenced by demographic characteristics of the exposed population and regional reporting differences. The higher proportion of reports involving female and elderly patients may reflect prescribing patterns rather than true differences in risk. Furthermore, the predominance of reports from the United States may introduce regional reporting bias and limit the generalizability of the findings.

## Figures and Tables

**Table 1:** Basic clinical features of the reports.

Characteristic		Amlodipine	Felodipine	Nicardipine	Nifedipine	Total
sex	male	13,585	247	472	1225	15,529
	female	15,967	361	731	2072	19,131
	unknown	3	0	1	0	4
Age group/years, y	y < 18	1112	5	101	225	1443
	18 ≤ y ≤ 44	3315	39	199	936	4489
	44 < y ≤ 64	9909	220	254	916	11,299
	64 < y ≤ 75	7252	161	263	574	8250
	y > 75	7967	183	387	646	9183
Report countries (Top Five)	United States of America	9242	58	211	1359	10,870
	United Kingdom of Great Britain and Northern Ireland	3133	282	2	145	3562
	Canada	4018	46	1	277	4342
	France	3471	31	821	65	4388
	Japan	1433	1	68	426	1928
Occupation of Reporters	Physician	16,072	351	725	1665	18,813
	Health Professional	8769	184	261	1250	10,464
	Pharmacist	4714	73	218	382	5387
Patient outcome	Other serious/important medical event	17,156	419	662	1907	20,144
	Hospitalization-Initial or Prolonged	14,909	276	614	1394	17,193
	Death	6687	59	92	657	7495
	Life-threatening	2978	99	178	316	3571
	Disability	780	64	6	40	890
	Congenital Anomaly	105	8	13	27	153
	Required Intervention to Prevent Permanent Impairment/Damage	42	0	1	6	49
	Unknown	1487	23	22	352	1884

Note: Since a single report may contain multiple adverse events, and each adverse event may correspond to multiple outcomes, the total number of adverse event outcome entries may exceed the number of reports.

**Table 2:** Top 20 PTs reported for amlodipine and felodipine in the context of the full dataset.

Amlodipine						Felodipine					
PT	Count	ROR	PRR	MHRA	IC	PT	Count	ROR	PRR	MHRA	IC
Completed suicide	3948	9.00 (8.71–9.29)	8.50	8.77; 26,351.72	3.04	Fatigue	210	1.58 (1.38–1.81)	1.37	1.57; 43.32	0.42
Toxicity to various agents	3371	4.36 (4.21–4.52)	4.14	4.28; 8383.01	2.02	Palpitations ^◆^	204	14.20 (12.36–16.31)	12.22	14.01; 2440.09	3.52
Hypotension ^▲^	3352	6.31 (6.10–6.54)	5.98	6.19; 14,277.52	2.54	Pyrexia	203	1.97 (1.72–2.27)	1.71	1.96; 94.92	0.73
Fall	1725	3.17 (3.03–3.33)	3.00	3.15; 2504.82	1.56	Abdominal discomfort	199	3.35 (2.91–3.85)	2.89	3.31; 319.93	1.48
Dyspnoea ^▲^	1661	1.38 (1.31–1.45)	1.31	1.38; 170.34	0.38	Headache ^◆^	199	2.05 (1.78–2.35)	1.77	2.03; 103.75	0.78
Acute kidney injury	1507	2.37 (2.25–2.49)	2.24	2.35; 1164.96	1.14	Migraine	197	8.93 (7.76–10.28)	7.67	8.82; 1355.25	2.87
Vomiting ^▲^	1189	1.14 (1.08–1.21)	1.08	1.14; 19.98	0.09	Pain	197	1.66 (1.44–1.91)	1.44	1.65; 50.44	0.49
Drug hypersensitivity	1164	1.39 (1.32–1.48)	1.31	1.39; 127.82	0.38	Tinnitus	195	31.31 (27.16–36.09)	26.84	30.88; 5544.18	4.59
Dizziness ^▲^	1160	1.60 (1.51–1.69)	1.50	1.59; 254.54	0.57	Dizziness ^◆^	175	2.48 (2.14–2.88)	2.13	2.46; 151.29	1.04
Bradycardia ^▲^	1057	6.26 (5.89–6.65)	5.85	6.22; 4518.83	2.50	Dyspnoea ^◆^	174	1.48 (1.28–1.72)	1.28	1.48; 26.67	0.31
Oedema peripheral	1048	3.38 (3.18–3.59)	3.16	3.36; 1716.79	1.63	Vomiting ^◆^	165	1.63 (1.40–1.90)	1.39	1.62; 39.08	0.44
Malaise ^▲^	962	1.23 (1.16–1.31)	1.16	1.23; 41.28	0.19	Nausea ^◆^	162	1.22 (1.05–1.43)	1.05	1.22; 6.24	0.03
Asthma	920	1.92 (1.79–2.04)	1.79	1.91; 395.71	0.82	Malaise	157	2.07 (1.77–2.43)	1.76	2.06; 85.20	0.77
Cough	882	1.89 (1.77–2.02)	1.77	1.89; 364.76	0.80	Chest pain ^◆^	152	4.40 (3.75–5.17)	3.73	4.37; 391.36	1.84
Orthostatic hypotension	841	8.98 (8.38–9.62)	8.34	8.93; 5716.22	2.99	Pruritus	151	2.13 (1.82–2.50)	1.81	2.12; 88.47	0.81
Suicide attempt	826	4.54 (4.24–4.87)	4.22	4.52; 2226.13	2.04	Dehydration	150	7.44 (6.33–8.74)	6.28	7.37; 818.44	2.58
Joint swelling ^▲^	804	1.14 (1.06–1.22)	1.06	1.13; 12.74	0.07	Diarrhoea ^◆^	150	1.31 (1.11–1.54)	1.11	1.31; 10.50	0.11
Asthenia ^▲^	755	1.11 (1.04–1.20)	1.04	1.11; 8.67	0.03	Angioedema ^◆^	149	13.06 (11.11–15.35)	11.01	12.93; 1620.80	3.36
Shock	739	12.02 (11.16–12.94)	11.11	11.96; 7076.82	3.39	Peripheral swelling	149	2.87 (2.44–3.38)	2.43	2.85; 177.75	1.23
Wheezing	736	1.65 (1.53–1.77)	1.53	1.64; 184.76	0.59	Back pain ^◆^	148	4.47 (3.80–5.26)	3.78	4.44; 390.83	1.86

Note: The ROR column in the table shows ROR values and their 95% confidence intervals (CI). The signal values for PRR and BCPNN methods correspond to the lower limit of the 95% CI, while MHRA signal values consist of (PRR; χ^2^). ^▲^ indicates mention in the amlodipine prescribing information; ^◆^ indicates mention in the felodipine prescribing information.

**Table 3:** Top 20 PTs reported for nicardipine and nifedipine in the context of the full dataset.

Nicardipine						Nifedipine					
PT	Count	ROR	PRR	MHRA	IC	PT	Count	ROR	PRR	MHRA	IC
Hypotension ^★^	104	8.61 (7.08–10.47)	6.92	8.36; 669.03	2.69	Maternal exposure during pregnancy	347	7.70 (6.92–8.57)	6.78	7.53; 1959.01	2.72
Acute kidney injury	87	6.15 (4.97–7.61)	4.88	6.01; 359.42	2.19	Completed suicide	326	7.46 (6.68–8.32)	6.56	7.30; 1767.66	2.67
Fall	68	5.57 (4.38–7.08)	4.32	5.47; 244.78	2.00	Premature delivery	276	48.49 (43.00–54.69)	42.25	47.53; 12,307.16	5.23
Hyperkalaemia	57	18.95 (14.58–24.63)	14.40	18.63; 932.52	3.57	Exposure during pregnancy	273	9.88 (8.77–11.15)	8.63	9.71; 2119.83	3.05
Maternal exposure during pregnancy	53	4.99 (3.80–6.54)	3.77	4.92; 162.07	1.79	Hypotension ^●^	268	5.11 (4.53–5.77)	4.47	5.03; 863.50	2.12
Bradycardia ^★^	52	13.54 (10.29–17.81)	10.18	13.34; 581.11	3.11	Toxicity to various agents	230	3.03 (2.66–3.45)	2.63	2.99; 304.40	1.36
Cerebral vasoconstriction	51	1361.91 (1016.54–1824.64)	1004.41	1340.03; 59,768.96	6.10	Drug hypersensitivity	186	2.32 (2.01–2.68)	2.00	2.30; 136.38	0.95
Eosinophilia	45	20.62 (15.36–27.69)	15.22	20.34; 807.56	3.57	Acute kidney injury	137	2.22 (1.87–2.63)	1.87	2.21; 89.45	0.85
Premature delivery	44	32.44 (24.08–43.70)	23.86	32.00; 1287.60	4.06	Shock	104	16.94 (13.96–20.56)	13.88	16.82; 1522.49	3.63
Cardiac arrest	41	9.10 (6.69–12.39)	6.64	9.00;283.64	2.52	Bradycardia	103	6.21 (5.11–7.54)	5.09	6.17; 440.26	2.26
Exposure during pregnancy	41	6.30 (4.63–8.57)	4.60	6.23; 175.14	2.03	Cardiac arrest	93	4.79 (3.91–5.87)	3.89	4.76; 272.67	1.88
Rash maculo-papular	37	15.95 (11.53–22.06)	11.45	15.77; 497.00	3.17	Oedema peripheral ^●^	91	3.01 (2.45–3.70)	2.44	3.00; 119.42	1.22
Acute generalised exanthematous pustulosis	34	45.25 (32.25–63.48)	32.03	44.77; 1406.30	4.20	Weight decreased	81	1.79 (1.44–2.23)	1.44	1.79; 27.26	0.46
Hyponatraemia	33	5.97 (4.24–8.42)	4.22	5.92; 130.27	1.88	Cough ^●^	73	1.62 (1.28–2.03)	1.28	1.61; 16.43	0.30
Premature baby	31	62.09 (43.55–88.53)	43.28	61.49; 1775.33	4.37	Dyspepsia ^●^	57	2.28 (1.76–2.96)	1.76	2.28; 39.55	0.73
Lactic acidosis	30	11.56 (8.07–16.57)	8.03	11.46; 276.10	2.68	Anuria	54	19.83 (15.16–25.93)	15.12	19.75; 935.77	3.61
Cholestasis	29	13.77 (9.55–19.85)	9.50	13.65; 327.28	2.87	Pulmonary oedema ^●^	51	6.06 (4.60–7.98)	4.59	6.04; 209.10	2.06
Drug reaction with eosinophilia and systemic symptoms	29	10.30 (7.15–14.86)	7.11	10.22; 232.07	2.52	Live birth	49	4.03 (3.04–5.34)	3.04	4.02; 108.13	1.49
Foetal exposure during pregnancy	28	21.80 (15.02–31.64)	14.95	21.62; 529.39	3.36	Hyperkalaemia	48	3.67 (2.76–4.87)	2.76	3.66; 89.97	1.35
Orthostatic hypotension ^★^	28	12.93 (8.91–18.76)	8.87	12.82; 293.41	2.78	Suicide attempt	47	2.64 (1.98–3.51)	1.98	2.63; 45.98	0.89

Note: The ROR column in the table shows ROR values and their 95% confidence intervals (CI). The signal values for PRR and BCPNN methods correspond to the lower limit of the 95% CI, while MHRA signal values consist of (PRR; χ^2^). ^★^ indicates mention in the nicardipine prescribing information; ^●^ indicates mention in the nifedipine prescribing information.

## Data Availability

The original contributions presented in the study are included in the article/Supplementary Materials. Further inquiries can be directed to the corresponding author.

## Supplementary Materials

The following supporting information can be downloaded at: https://www.mdpi.com/article/10.3390/ph19040544/s1, Table S1: Calculation Formulas and Signal Judgment Criteria for Four Types of Proportion Imbalance Methods; Table S2: List of DHP-CCBs drugs; Table S3: Multivariate Logistic Regression Analysis of Suicide Risk Associated with DHP-CCBs; Figure S1: Forest plot of ROR signal detection for sensitivity analysis of suicide-related adverse events associated with amlodipine in the full dataset; Figure S2: Forest plot of ROR signal detection for sensitivity analysis of pregnancy-related adverse events associated with nicardipine and nifedipine in the full dataset; Figure S3: Forest plot of ROR signal detection for sensitivity analysis by reporter type in the full dataset.
