# Supplementary material for: Signal Detection of Adverse Events Associated with Four Dihydropyridine Calcium Channel Blockers Based on the FAERS Database
Source: Pharmaceuticals (Basel). 2026 Mar 28;19(4):544. doi: 10.3390/ph19040544 (PMC13118446; doi:10.3390/ph19040544)
Supplement: Supplementary file 1 [file pharmaceuticals-19-00544-s001.zip › pharmaceuticals-4168092-supplementary.pdf]

Signal [M2.1]Detection of Adverse Events Associated with Four Dihydropyridine Calcium Channel Blockers Based on the FAERS Database

Journal name: Pharmaceuticals

Authors

Zicong Guo, Yi Guo, Xiaoxiao Quan, Rui Xiao, Jia Li and Wei Liu

Affiliations

School of Pharmaceutical Sciences, Zhengzhou University, No. 100 Science Avenue, High-Tech Zone, Zhengzhou 450001, China — E-mails: guozicongyxy@gs.zzu.edu.cn; 174455877@qq.com; 462255781@qq.com; xiaoyyy@gs.zzu.edu.cn; li15837016326@gs.zzu.edu.cn; liuweiyxy@zzu.edu.cn

Corresponding Author

Wei Liu

School of Pharmaceutical Sciences, Zhengzhou University, No. 100 Science Avenue, High-Tech Zone, Zhengzhou 450001, China.

E-mail: liuweiyxy@zzu.edu.cn.

Tel: +86-138-3800-5960.

Table S1 List of DHP-CCBs drugs

	ATC Code	Drug
C08CA Dihydropyridine derivatives	C08CA01	amlodipine
	C08CA02	felodipine
	C08CA03	isradipine
	C08CA04	nicardipine
	C08CA05	nifedipine
	C08CA06	nimodipine
	C08CA07	nisoldipine
	C08CA08	nitrendipine
	C08CA09	lacidipine
	C08CA10	nilvadipine
	C08CA11	manidipine
	C08CA12	barnidipine
	C08CA13	lercanidipine
	C08CA14	cilnidipine
	C08CA15	benidipine
	C08CA16	clevidipine
	C08CA17	levamlodipine

Table S2 Multivariate Logistic Regression Analysis of Suicide Risk Associated with DHP-CCBs

Variable	Number of Reports	Completed suicide		Suicide attempt	
		Adjusted OR (95%CI)	p	Adjusted OR (95%CI)	p
Sex					
female	18858	1.35（1.26-1.44）	7.6×10^{-19}	0.69（0.60-0.78）	1.7×10^{-8}
male	15344	1.00（Reference)		1.00（Reference)	
Age group (years)					
＜18	1404	0.17（0.13-0.21）	4.5×10^{-41}	1.33（1.04-1.68）	2.0×10^{-2}
18-44	4399	1.00（Reference)		1.00（Reference)	
45-64	11126	0.98（0.90-1.08）	0.7	0.57（0.48-0.67）	2.8×10^{-11}
65-75	8172	0.47（0.43-0.53）	1.1×10^{-41}	0.30（0.24-0.37）	7.3×10^{-30}
＞75	9105	0.28（0.25-0.32）	1.7×10^{-97}	0.11（0.08-0.15）	4.7×10^{-50}
Occupation					
Physician	18544	1.00（Reference)		1.00（Reference)	
Pharmacist	5362	1.22（1.11-1.33）	1.7×10^{-5}	0.67（0.49-0.89）	8.4×10^{-3}
Health Professional	10300	0.08（0.07-0.09）	9.3×10^{-310}	0.83（0.72-0.94）	5.0×10^{-3}
Drugs					
amlodipine	29555	1.00（Reference)		1.00（Reference)	
felodipine	608	0.06（0.04-0.10）	1.3×10^{-30}	0.08（0.03-0.16）	1.6×10^{-10}
nicardipine	1204	0.0027（0-0.0185）	3.0×10^{-5}	0.19（0.06-0.43）	5.0×10^{-4}
nifedipine	3297	0.78（0.69-0.88）	5.9×10^{-5}	0.44（0.32-0.58）	3.3×10^{-8}

Note: In terms of completed suicide, compared with amlodipine (reference group), felodipine was

associated with a 94% reduced reported risk (adjusted OR = 0.06, 95% CI: 0.04-0.10, P < 0.01), nicardipine with a 99.7% reduction (adjusted OR = 0.0027, 95% CI: 0-0.0185, P < 0.01), and nifedipine with a 22% reduction (adjusted OR = 0.78, 95% CI: 0.69-0.88, P < 0.01).

In terms of suicide attempt, again using amlodipine as the reference, felodipine was associated with a 92% reduced reported risk (adjusted OR = 0.08, 95% CI: 0.03-0.16, P < 0.01), nicardipine with an 81% reduction (adjusted OR = 0.19, 95% CI: 0.06-0.43, P < 0.01), and nifedipine with a 56% reduction (adjusted OR = 0.44, 95% CI: 0.32-0.58, P < 0.01).

Table S3 Calculation Formulas and Signal Judgment Criteria for Four Types of Proportion Imbalance Methods

Signal Detection Method	Formula	Signal Evaluation Criteria
ROR	ROR = (a/b)/(c/d) = ad/bc<br>95%CI = $e^{\ln(ROR)\pm 1.96\sqrt{\frac{1}{a}+\frac{1}{b}+\frac{1}{c}+\frac{1}{d}}}$	N≥3，95%CI＞1
PRR	PRR=(a/(a+b))/(c/(c+d))<br>95%CI = $e^{\ln(PRR)\pm 1.96\sqrt{\frac{1}{a}-\frac{1}{(a+b)}+\frac{1}{c}-\frac{1}{(c+d)}}}$	N≥3，95%CI＞1
MHRA	$x^2 = \frac{N*(\lvert a*b-d*c\rvert-\frac{N}{2})^2}{(a+b)*(c+d)*(a+c)*(b+d)}$	N≥3，PRR≥4，x^2≥4
IC	IC = $\log_2\frac{a+1/2}{E+1/2}$<br>$IC95\%CI_{lower} = \log_2\frac{a+1/2}{E+1/2} - 3.3\times(a+1/2)^{-1/2} - 2\times(a+1/2)^{-3/2}$	N≥3，IC95%CI $_{lower}$ ＞0

Note: a: Target ADE count for the target drug; b: Other ADE count for the target drug; c: Target ADE count for other drugs; d: Other ADE count for other drugs; N: Number of reports. CI: Confidence Interval.

DRUG	PT	N	ROR(95% CI)
**Before sensitivity analysis**			
Amlodipine	Completed suicide	3948	9.00 (8.71, 9.29)
Amlodipine	Suicide attempt	826	4.54 (4.24, 4.87)
**After sensitivity analysis**			
Amlodipine	Completed suicide	2334	7.43 (7.13, 7.74)
Amlodipine	Suicide attempt	677	4.96 (4.59, 5.35)

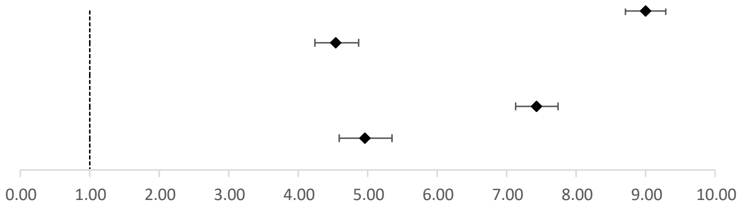

Figure S1 Forest plot of ROR signal detection for sensitivity analysis of suicide-related adverse events associated with amlodipine in the full dataset

Before sensitivity analysis

DRUG	PT	N	ROR(95%CI)
Nicardipine	Maternal exposure during pregnancy	53	4.99 (3.80, 6.54)
Nicardipine	Premature delivery	44	32.44 (24.08, 43.70)
Nicardipine	Exposure during pregnancy	41	6.30 (4.63, 8.57)
Nifedipine	Maternal exposure during pregnancy	347	7.70 (6.92, 8.57)
Nifedipine	Premature delivery	276	48.49 (43.00, 54.69)
Nifedipine	Exposure during pregnancy	273	9.88 (8.77, 11.15)

After sensitivity analysis

DRUG	PT	N	ROR(95%CI)
Nicardipine	Maternal exposure during pregnancy	5	8.04 (3.32,19.42)
Nicardipine	Premature delivery	11	74.77 (41.01, 136.31)
Nicardipine	Exposure during pregnancy	13	30.83 (17.72, 53.63)
Nifedipine	Maternal exposure during pregnancy	28	6.22 (4.28, 9.03)
Nifedipine	Premature delivery	33	30.65 (21.72, 43.26)
Nifedipine	Exposure during pregnancy	49	15.86 (11.95, 21.05)

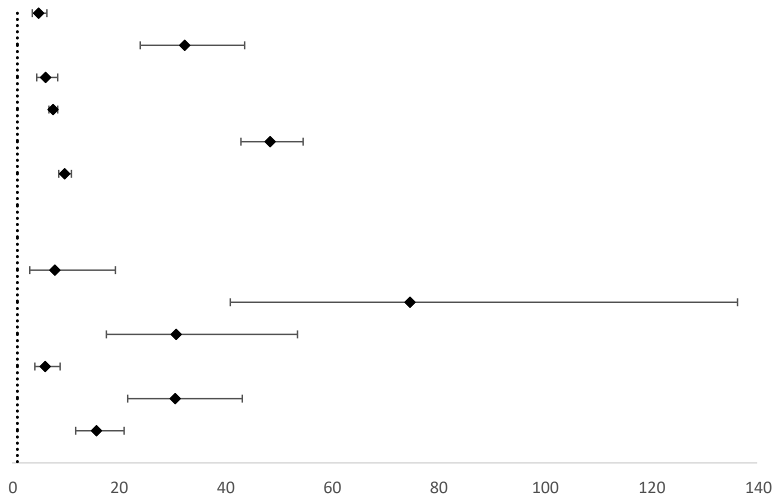

Figure S2 Forest plot of ROR signal detection for sensitivity analysis of pregnancy-related adverse events associated with nicardipine and nifedipine in the full dataset

Before sensitivity analysis

DRUG	PT	N	ROR(95%CI)
Amlodipine	Completed suicide	3948	9.00 (8.71, 9.29)
Amlodipine	Suicide attempt	826	4.54 (4.24, 4.87)
Felodipine	Palpitations	204	14.20 (12.36, 16.31)
Felodipine	Angioedema	149	13.06 (11.11, 15.35)
Nicardipine	Acute generalised exanthematous pustulosis	34	45.25 (32.25, 63.48)
Nifedipine	Premature delivery	276	48.49 (43.00, 54.69)
Nifedipine	Exposure during pregnancy	273	9.88 (8.77, 11.15)

After sensitivity analysis

DRUG	PT	N	ROR(95%CI)
Amlodipine	Completed suicide	3029	9.90 (9.54, 10.27)
Amlodipine	Suicide attempt	404	3.61 (3.27, 3.98)
Felodipine	Palpitations	84	4.89 (3.95, 6.07)
Felodipine	Angioedema	81	29.14 (23.38, 36.33)
Nicardipine	Acute generalised exanthematous pustulosis	31	47.62 (33.35, 67.99)
Nifedipine	Premature delivery	78	35.57 (28.41, 44.54)
Nifedipine	Exposure during pregnancy	107	12.80 (10.57, 15.51)

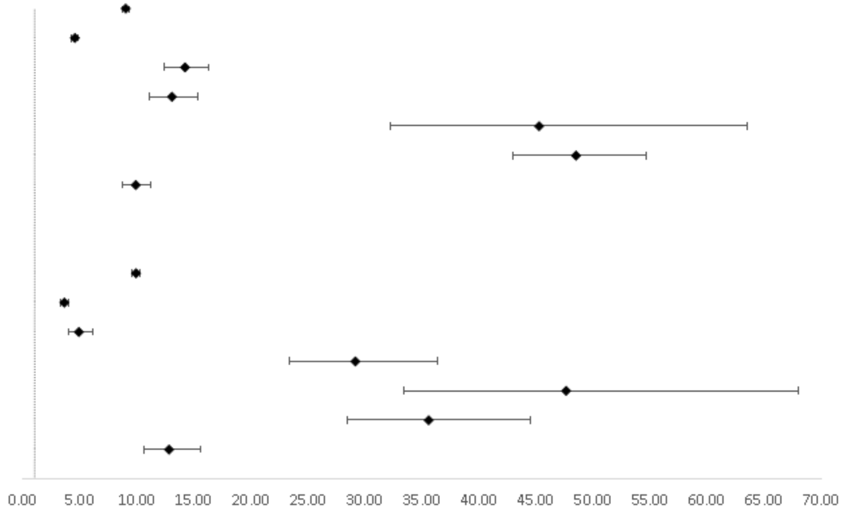

Figure S3 Forest plot of ROR signal detection for sensitivity analysis by reporter type in the full dataset

Note: In the sensitivity analysis stratified by reporter type, we also analyzed the signal of cerebral vasoconstriction associated with nicardipine.Before sensitivity analysis, the ROR was 1361.91 (95%CI: 1016.54, 1824.64) with 51 reports; after sensitivity analysis, the ROR was 1486.12 (95%CI: 1097.85, 2011.71) with 50 reports.Since this signal value was extremely large, its inclusion in the forest plot would result in severe overlapping of other signal intervals; therefore, it was not displayed in the figure.
